# Clinical Efficacy of Endovenous Radiofrequency Ablation (RFA) for Superficial Varicose Veins of the Lower Extremities

**DOI:** 10.1155/2022/1673588

**Published:** 2022-06-20

**Authors:** Wei Jia, Jianlong Liu, Zhiyuan Cheng

**Affiliations:** Department of Vascular Surgery, Beijing Jishuitan Hospital, Beijing 100035, China

## Abstract

**Objective:**

To investigate the clinical efficacy of intravenous radiofrequency ablation (RFA) in the treatment of patients with superficial varicose veins of lower extremities.

**Methods:**

From January 1, 2021, to January 1, 2022, 62 patients with superficial lower extremity varicose veins were selected and divided into two groups according to the treatment plan. 31 patients underwent high saphenous vein ligation and dissection as control. Thirty-one patients received RFA treatment as the experimental group. The operation-related indicators, clinical efficacy, and postoperative complications were compared.

**Results:**

The intraoperative blood loss in the experimental group was significantly less than that in the control group. The clinical efficacy of the experimental group was significantly better than that of the control group. The incidence of postoperative complications in the experimental group was lower than that in the control group.

**Conclusion:**

RFA has a good clinical effect in the treatment of patients with superficial lower extremity varicose veins, with less postoperative complications, and has a high therapeutic value.

## 1. Introduction

Superficial varicose veins of the lower extremities are a common clinical condition, caused by blood reflux and stagnation due to the dysfunction of the superficial veins of the lower extremities, the abnormal structure of the vein walls, and venous hypertension main causes [1]. Most patients have no obvious discomfort, but some patients may experience soreness and swelling of the legs, and in severe cases, complications such as ulceration, bleeding, and superficial phlebitis may occur [2]. Surgery is widely used in the clinical treatment of superficial varicose veins in the lower extremities and is the only curable option, which has the advantage of being curative compared to conservative treatment, but surgery has certain risks and the possibility of recurrence after surgery [3]. The traditional procedure with a high prevalence rate is saphenous vein ligation and stripping, which has a small incision and ideal efficacy [4]. In recent years, with the continuous improvement of clinical medical level, the application of endovenous radiofrequency ablation has gradually increased and obtained good efficacy with the advantages of minimally invasive and fast postoperative recovery [5]. In this study, 62 patients with superficial varicose veins of lower limbs from January 2021 to January 2022 were selected for comparative analysis to investigate the clinical efficacy of RFA for superficial varicose veins of lower limbs.

## 2. Methods

### 2.1. Patients

A total of 68 patients with superficial varicose veins of the lower extremities were selected for the study from January 2021 to January 2022. Patients and family members of both groups gave informed consent to this study and signed the informed consent form; the study was conducted after review and approval by the Medical Ethics Committee.

Inclusion criteria are as follows: (1) swelling and pain in the affected limbs, confirmed by imaging and physical examination; (2) complete medical records; and (3) good mental status. Exclusion criteria are as follows: (1) contraindication to surgery or anesthesia, (2) coagulation dysfunction, (3) women during pregnancy and lactation, and (4) psychiatric diseases.

### 2.2. Interventions

Patients in the control group were provided with preoperative education about surgical options, risks, costs, and consented. Ultrasound is performed on the patient to mark the location of the main saphenous vein and varices. The patient underwent high saphenous vein ligation and dissection. The patient received general anesthesia in the supine position and underwent routine disinfection and towel wiping. A surgical incision was made at 1 cm medial to the inguinal inferior femoral artery pulse; the skin, subcutaneous tissue, and fascia were incised layer by layer; the trunk of the great saphenous vein and the geniculate branch were freed, and each geniculate branch was ligated. The main trunk of the great saphenous vein at 0.5 cm from the opening of the femoral vein was clipped, and the proximal double ligation was disconnected. A surgical incision was made at the trunk of the great saphenous vein on the medial side of the knee joint, the skin and subcutaneous tissue were incised layer by layer, the trunk of the great saphenous vein was dissociated and disconnected, and the metatarsal bones were double ligated. The dissector is inserted proximally into the groin. The distal end of the dissector is ligated and fixed with the trunk of the great saphenous vein. The main trunk of the great saphenous vein was dissected proximally, and pressure was applied to stop the bleeding for 10 minutes. Postoperatively, an elastic bandage is applied to the patient with a compression bandage.

For patients in the experimental group, the preoperative preparations were the same as those in the control group. Intracavitary radiofrequency ablation was performed. The patient was anesthetized by local infiltration, taken in the supine position, routinely disinfected, and wiped with a towel. A 21G needle was used to puncture the main trunk of the great saphenous vein (middle and upper third of the calf) under ultrasound guidance. A short 6F sheath is placed in the trunk of the great saphenous vein using a guidewire, and the guidewire and sheath core are removed. An endoluminal radiofrequency ablation catheter and a guide wire (0.018 inch) were placed into the trunk of the great saphenous vein under ultrasound guidance. Ultrasound positioning was performed, and the catheter tip was locked at 2 cm from the saphenofemoral junction. A tumescent anesthetic solution is injected along the area lining the great saphenous vein. The energy generator was started, radiofrequency treatment was performed for 20 s/time, the temperature of the great saphenous vein was raised to 120°C, and the treatment was repeated once in the initial segment. The radiofrequency ablation closure catheter and short sheath are removed after the injury and compression bandaged. Postoperatively, the patient wore compression stockings. The surgeries for patients in two groups were performed by the same group of trained and experienced physicians.

### 2.3. Observation Indicators

The surgical indicators, clinical efficacy, and postoperative complications were compared. Surgical indicators included operative time and intraoperative blood loss. The surgical efficacy was based on the venous clinical severity score (VCSS), with a full score of 30 points. Reduction rate > 70% is significantly effective, reduction rate 30-70% is effective, and reduction rate < 30% is considered ineffective. Efficacy = (significantly effective + effective)/number of cases × 100%. Postoperative complications include wound infection, phlebitis, and saphenous nerve injury.

### 2.4. Statistical Analysis

The SPSS 25.0 statistical software (IBM, USA) was used to analyze the measurement data; the data were expressed as mean ± sd. The differences were determined by *t*-test and *χ*^2^ test; the two-sided *P* less than 0.05 was considered significant differences.

## 3. Results

### 3.1. Comparison of Surgical Indexes between the Two Groups

A total of 68 patients were enrolled in this study. Six patients were excluded based on inclusion and exclusion criteria. According to the different groups of the surgical protocol, 62 patients were divided into two groups. There were 31 cases in the control group, 16 males and 15 females; ages were 37-78 years, with mean of 64.34 ± 4.05 years. The duration of disease was 6 months to 2 years, with mean of 1.21 ± 0.18 years. The affected limbs were 10 cases on the left side and 21 cases on the right side. In the experimental group, there were 31 cases, 17 males and 14 females; ages were 36-78 years, with an average of 64.29 ± 4.01 years. The duration of disease was 6 months to 2 years, with an average of 1.20 ± 0.16 years. The affected limbs were 12 cases on the left side and 19 cases on the right side. As shown in Table 1, the intraoperative bleeding of patients in the test group was significantly less than that of the control group.

### 3.2. Comparison of Clinical Efficacy between the Two Groups

As shown in Table 2, the clinical efficacy of the test group was significantly better than that of the control group (*P* < 0.05), indicating that RFA can effectively treat superficial lower extremity varicose veins.

### 3.3. Comparison of the Incidence of Postoperative Complications between the Two Groups

The incidence of postoperative complications in the experimental group was significantly lower than that in the control group (*P* < 0.05), indicating that RFA can significantly reduce postoperative complications (Table 3).

## 4. Discussion

Superficial varicose veins of the lower extremities have a high clinical incidence and are common vascular lesions, mainly due to weakness of the superficial vein wall and poor structure and function of the venous valves [6]. Long-term standing and heavy physical work are the main triggering factors, and most patients have no obvious symptoms, but a few may experience soreness and discomfort in both legs [7]. Clinically, the treatment of superficial varicose veins in lower limbs can be compression therapy, medication, and surgery, among which compression therapy and medication are conservative treatments, which have a relieving effect on the symptoms of the disease and slow down the progress of the disease but cannot cure it, and the symptoms worsen with time [8]. Surgery is the only option to cure superficial varicose veins in the lower extremities, and choosing the right surgical option is crucial to improve clinical outcomes and prognosis [9].

In this study, patients in the control group were treated with high saphenous vein ligation and stripping, and patients in the trial group were treated with endovenous radiofrequency ablation. When comparing the two surgical protocols, the operative time was the same, and the intraoperative bleeding was less with endovenous radiofrequency ablation than with high saphenous vein ligation and stripping. Because endovenous radiofrequency ablation is a minimally invasive procedure, the surgical incision is small, and the operation is simple [10]. After years of improvement, the incision of saphenous vein ligation and stripping has been reduced compared with the previous one, but the surgery is still more traumatic, with many postoperative complications, slow recovery, and long hospital stay [11, 12]. The clinical efficacy of the experimental group is better than that of the control group. The reasons for this are as follows: endovenous radiofrequency ablation, as a minimally invasive procedure, has a small surgical incision compared with high saphenous vein ligation and stripping, and the surgical operation is performed under ultrasound guidance, with accurate localization of the saphenous vein and high operational refinement [13, 14]. Patients do not need to stay in bed for a long time to recover after surgery; they can get out of bed immediately after recovery from anesthesia and recover quickly after surgery [15]. No postoperative complications occurred in the test group, and the rate of postoperative complications was low compared to the control group. Analysis of the reason: two surgical incisions are needed for saphenous vein ligation and stripping, while only one surgical incision is made for endovenous radiofrequency ablation, which reduces the difficulty of postoperative care by reducing one surgical incision and makes the incision treatment easy and less prone to infection [16, 17]. Intracavitary radiofrequency ablation is performed intravenously during the operation, and the operation is precise under ultrasound guidance, with little damage to the surrounding tissues [18, 19]. Endovenous radiofrequency ablation performs local infiltration anesthesia, while saphenous vein high ligation and stripping requires general anesthesia, which has a high anesthetic impact and is not conducive to postoperative recovery, long bed rest, and high risk of complications [20].

In this study, the intraoperative bleeding in the test group was less than that in the control group, and the operative time was comparable. The efficacy of the test group was better than that of the control group, and the postoperative complication rate was lower than that of the control group. This indicates that the overall results and postoperative recovery of endovenous radiofrequency ablation for the treatment of superficial varicose veins in the lower extremities are better compared with high saphenous vein ligation and stripping.

In conclusion, the clinical efficacy of endovenous radiofrequency ablation for the treatment of superficial varicose veins in the lower extremities is precise and worthy of application.

## Figures and Tables

**Table 1:** Comparison of surgical indicators between the two groups.

Group	Surgery time (mins)	Intraoperative bleeding (mL)
Test group (*n* = 31)	62.15 ± 8.45	19.24 ± 2.45
Control group (*n* = 31)	62.54 ± 8.34	33.05 ± 3.15
*t*	0.183	19.268
*P*	0.855	0.001

**Table 2:** Comparison of clinical efficacy between the two groups.

Group	Significantly effective	Effective	Invalid	Efficient
Test group (*n* = 31)	14 (45.16%)	15 (48.39%)	2 (6.45%)	29 (93.55%)
Control group (*n* = 31)	10 (32.26%)	13 (41.94%)	8 (25.81%)	23 (74.19%)
*χ* ^2^				4.292
*P*				0.038

**Table 3:** Comparison of the incidence of postoperative complications between the two groups.

Group	Incision infection	Phlebitis	Saphenous nerve injury	Incidence
Test (*n* = 31)	1 (3.23%)	1 (3.23%)	1 (3.23%)	3 (9.67)
Control (*n* = 31)	7 (22.58%)	2 (6.46%)	3 (9.67%)	12 (38.71%)
*χ* ^2^				6.781
*P*				0.009

## Data Availability

We confirm that all data were included in the manuscript.
